# The CHIR Score for Evaluating the Hyperimmune Response in COVID-19: A Preliminary Concept

**DOI:** 10.3389/fpubh.2020.550933

**Published:** 2020-09-22

**Authors:** Daniel Kumar Goyal, Fatma Mansab

**Affiliations:** ^1^Gibraltar COVID-19 Research Group, University of Gibraltar, Gibraltar, United Kingdom; ^2^Acute General Medicine, Department of Medicine, St Bernard's Hospital, Gibraltar Health Authority, Gibraltar, United Kingdom; ^3^Health Advisory Unit, Public Health Gibraltar, Gibraltar, United Kingdom

**Keywords:** COVID-19, SARS-CoV-2, 2019-nCov, hyperimmune, cytokine storm, corticosteroids, treatment, inflammation

## Abstract

Similar to SARS and MERS, the host immune response to COVID-19 is implicated in the severity of the disease itself. Here, we investigate the possible use of scoring systems to help guide clinicians in their determination as to when to commence immunosuppressive treatment in COVID-19. We utilized the relatively established clinical and biochemical severity indicators from large cohort studies to develop a potential scoring system for the hyperimmune response in COVID-19.

## Introduction

SARS-CoV2 causes COVID-19 (Coronavirus Disease 2019). As of April 2020, the total reported cases of COVID-19 was over 1.5 million, with 100,000 deaths over 4 months. More than half these deaths have occurred in the last month.

Severity of COVID-19 can be associated with prolonged fever, rising inflammatory markers and signs of systemic inflammation such as bone marrow suppression, in the absence of secondary infection (1). A growing body of evidence suggests such an inflammatory response may be more related to a hyperimmune response as opposed to the direct effects of the virus.

Total viral load in COVID-19 seems to decrease after the initial phase of the infection (2), and early evidence suggests such a reduction in viral load does not correlate with severity or mortality outcomes (3, 4), although there is a report of correlation between stage of disease (early, progressive, and recovery) and level of virus in nasopharyngeal swabs (5). It seems individuals with low viral loads can still progress to severe lung pathology, and equally, those with similar viral loads can suffer only mild symptoms and recover quickly (5).

Postmortem examinations have identified marked inflammatory changes within the lung tissue, together with virally activated T-cells without intranuclear or intracytoplasmic viral inclusions. This led investigators to suggest that based on the pathological findings in lung tissue, immunosuppressive medication would be indicated (6).

Such a hyperimmune response can be characterized in a number of ways. Cytokine Release Syndrome (CRS), Haemophagocytic Syndrome (HS), and Cytokine Storm Syndrome (CSS) have all been described in COVID-19 (7, 8). Commonalities between these different hyperimmune responses include, systemic upset with pyrexia and malaise; rising inflammatory markers with bone marrow suppression, and eventually Systemic Inflammatory Response Syndrome (SIRS) and Acute Respiratory Distress Syndrome (ARDS).

Similar findings were discovered in SARS and MERS (9, 10).

Most of these hyperimmune states respond to disease modifying agents. For example, Cytokine Release Syndrome is often responsive to Tocilizumab or corticosteroids (11). Haemophagocytic Syndrome is managed with corticosteroids, intravenous Immunoglobulin or Tocilizumab (8).

### Disease Modifying Agents in SARS-Cov2

SARS treatment was developed during the outbreak itself, based on clinical findings and experience. Over time, the mainstay of treatments included ribavarin, broad-spectrum antibiotics and corticosteroids. Such practice became established. This led to challenges in undertaking Randomized Contrlled Trials (RCT). As such, the evidence for these established interventions lacks the power to provide the certainty necessary to generate national or international guidelines (12). Still to this day, many different groups maintain different protocols for the treatment of SARS, although corticosteroids remain a cornerstone of intervention.

There is limited data on corticosteroid use in SARS-CoV2. One of the few studies report on a retrospective analysis of low-dose, short-term corticosteroid use in patients with severe COVID-19 infection. They report significant improvement in oxygenation and resolution of CT changes with 5–7 days of 1–2 mgs/kg/day of methylprednisolone vs. no methylprednisolone (8.2 days [IQR 7.0–10.3] vs. 13.5 days [IQR 10.3–16]; *P* < 0.001). There were only three deaths in the total cohort of 46, so no inference relating to mortality can be drawn (13).

A further trial is underway in China with a dosing schedule of 1–2 mgs/kg methylprednisolone for a duration of 3 days (14).

There are additional trials currently underway examining other disease modifying agents in COVID-19, including Tocilizumab, Immunoglobulin, and Convalescent Plasma.

## Generating a Hyperimmune Scoring System

In this present study we aimed to generate an initial, testable hyperimmune score specific to COVID-19. Building on the suggestions of Cron et al. (7) and Mehta et al. (8), and with the increasing body of evidence supporting various inflammatory markers as disease severity indicators (15–17), we first took the validated hyperimmune score associate with hyperphagocytic syndrome, the HScore, and adapted it to COVID-19 specific clinical features (Table 1).

**Table 1:** COVID-19 Hyperimmune Response (CHIR) scoring criteria.

**Measure**	**Points**
**Temperature (C)**	
38.4–39.4	30
>39.4	50
**Days of symptoms**	
3–7	30
7–10	15
**White cell count (10**^**9/**^**L)**	
<6.0	30
**Lymphocytes (10**^**9/**^**L)**	
<1.0	15
<0.5	30
**AST (IU/L)**	
>30	15
**Platelets (10**^**9/**^**L)**	
<110	15
<90	30
<60	50
**CRP (mg/L)**	
>100	15
>200	30
**Procalcitonin (ng/ml)**	
<0.21	15
>0.5 and <0.8	−25
0.8 to 1.0	−50
>1.0	−75
**Total score**	**Hyperimmune Response**
<80	Unknown
80–149	Possible
>149	Likely

*WCC, White Cell Count; CRP, C-Reactive Protein; PCT, Procalcitonin; AST, Aspartate Aminotransferase*.

COVID-19 pneumonia typically presents with high CRP, relatively low PCT and often low lymphocytes (16, 17). The persistence of fever, further bone marrow suppression (e.g., thrombocytopenia)—in the absence of evidence for secondary infection—would be typically interpreted as a systemic inflammatory response. The COVID-19 Hyperimmune Score (CHIR Score) was designed to reflect these relative consistencies in clinical and pathological parameters. Crucially, the scoring system was designed to aid in the *confirmation* of a hyperimmune response, and *not* to have any negative predictive value (i.e., the CHIR Score cannot provide any guidance as to the absence of a hyperimmune response). As such scores were divided into Likely (Score 150 and above) and Possible (Score 80–150).

In determining the weighting for each clinical or pathological parameter we considered point measures with ease of repeating.

Fever was given a relatively substantive weighting. Whilst a hyperinflammatory syndrome can occur without fever (particularly in the elderly), the presence of fever is the most common symptom (11). There is limited evidence for the relationship between level of fever and severity of hyperimmune response, however the severity of fever is generally viewed as a marker of severity. As such, the CHIR score attributes a greater value to temperatures over 39.4°C.

The “Days to Onset” may not determine the probability of a hyperimmune response, but those who develop marked inflammatory changes early in the disease (in the absence of secondary infection) seem to be at risk of a more severe inflammatory cascade, consistent with other hyperinflammatory syndromes (7). As such the CHIR score attributes a higher score to a shorter history.

White cell count, lymphocyte count and platelet count are attributed a positive score at varying levels in the CHIR score due to the frequent occurrence of bone marrow suppression in an acute hyperinflammatory syndrome (11, 18), and the quite consistent relationship between level of lymphopenia with severity (16, 17).

C-Reactive Protein (CRP) is a marker of inflammation, and as such has relevance in a hyperinflammatory condition. As it is a non-specific marker of inflammation—rising in infection, a hyperimmune response and malignancy -, it is not discriminatory between infection and inflammation, and as such is attributed a modest predictive score.

Evidence suggests procalcitonin (PCT) has discriminatory value between infective inflammation and non-infective inflammation (19). A significantly raised PCT is highly suggestive of bacterial infection. Given the main differential when considering a hyperinflammatory state is a secondary bacterial infection, PCT has added value, and has been attributed a high negative CHIR score.

The CHIR Score is only intended to be utilized where there is confidence that a secondary infection has been excluded. Patients with, for example, clear and confirmed immunosuppression (e.g., neutropenia) may well-achieve higher CHIR scores, but may be more likely to have superadded infection vs. a hyperimmune response. The negative scoring of significantly raised PCT serves to mitigate such patient groups, however clinical acumen remains a necessity in interpretation.

## Discussion

An international collaboration led by the American Thoracic Society recently issued emergency guidance on treatment recommendations in COVID-19. This pragmatic stance reflects the reality of the significant time-lag to the results of sufficiently powered RCTs, and the need for treatment options during the pandemic (20).

Physicians on the ground facing this new disease must make the best decisions they can based on their knowledge, experience, and the limited available data. Hyperimmune scoring systems such as the CHIR Score presented here may provide some support in the consideration of when to commence disease modifying agents or immunosuppressives such as corticosteroids in patients with severe COVID-19. As such, this publication builds on the suggestion made by Zhou et al. (21): tailored and responsive corticosteroids may well-offer survival benefit in SARS-CoV2.

The CHIR Score remains a preliminary concept. Future studies will include a retrospective analysis of the predictive power of the CHIR Score in determining treatment responsiveness in clinical trials involving immunosuppressive medications. If predictive, a prospective clinical trial would be required to validate the score as a usable clinical tool.

## Data Availability Statement

Publicly available datasets were analyzed in this study. This data can be found here: https://www.ncbi.nlm.nih.gov/pmc/articles/PMC7143164/bin/NEJMoa2004500_appendix.pdf.

## Author Contributions

All authors contributed to the concept, design, write-up, and final approval of the article.

## Conflict of Interest

The authors declare that the research was conducted in the absence of any commercial or financial relationships that could be construed as a potential conflict of interest.

## Abbreviations

SARS: Severe Acute Respiratory Syndrome
COVID-19: Coronavirus Disease 2019
SARS-CoV2: Severe acute respiratory syndrome coronavirus 2
MERS: Middle East Respiratory Syndrome.
